# Viral Inhibition of the IFN-Induced JAK/STAT Signalling Pathway: Development of Live Attenuated Vaccines by Mutation of Viral-Encoded IFN-Antagonists

**DOI:** 10.3390/vaccines4030023

**Published:** 2016-06-29

**Authors:** Stephen B. Fleming

**Affiliations:** Department of Microbiology and Immunology, University of Otago, 720 Cumberland St, Dunedin 9016, New Zealand; stephen.fleming@otago.ac.nz; Tel.: +64-3-4797727; Fax: +64-3-4797744

**Keywords:** interferon, viruses, immune evasion, JAK/STAT pathway, vaccines, innate immunity

## Abstract

The interferon (IFN) induced anti-viral response is amongst the earliest and most potent of the innate responses to fight viral infection. The induction of the Janus kinase/signal transducer and activation of transcription (JAK/STAT) signalling pathway by IFNs leads to the upregulation of hundreds of interferon stimulated genes (ISGs) for which, many have the ability to rapidly kill viruses within infected cells. During the long course of evolution, viruses have evolved an extraordinary range of strategies to counteract the host immune responses in particular by targeting the JAK/STAT signalling pathway. Understanding how the IFN system is inhibited has provided critical insights into viral virulence and pathogenesis. Moreover, identification of factors encoded by viruses that modulate the JAK/STAT pathway has opened up opportunities to create new anti-viral drugs and rationally attenuated new generation vaccines, particularly for RNA viruses, by reverse genetics.

## 1. Introduction

Viruses are obligate parasites that replicate within living cells and use the host’s cellular machinery to do this. During the course of evolution the host has evolved highly sophisticated mechanisms to circumvent infection by viruses and to rapidly shut down their ability to replicate. IFNs are one of the most important weapons in the host’s arsenal to resist infection by viruses and induce an antiviral response through the induction of a vast array of genes in host cells that impede viral replication. Although type 1 IFNs were discovered about 50 years ago, it has only been in the last 15–20 years that it has come to light that many viruses have evolved mechanisms to counteract such a potent host innate response [1]. Induction of the Janus kinase/signal transducer and activation of transcription (JAK/STAT) signalling pathway by IFNs gives rise to the expression of interferon stimulated genes (ISGs). The identification and characterisation of the signalling components of this pathway paved the way for the discovery of strategies by which viruses manipulate IFN signalling [2,3]. Almost all cells in mammalian and avian hosts produce IFNs and have the ability to respond to IFNs through the engagement of IFN cellular receptors that induce the IFN effector response via the JAK/STAT signalling pathway [4]. Since the induction of the antiviral response by IFN is a major threat to virus survival, the JAK/STAT pathway has become a target for attack by viruses and these microorganisms have evolved a myriad of ways to do this.

IFNs are a multifunctional family of cytokines that play a critical role as a first line of defence against viral infection [5,6]. Type 1 IFNs α/β are induced in virus infected cells by engagement of viral molecules, in particular nucleic acids, with pattern recognition receptors and induce innate immune responses [7]. Most human cell types can produce type 1 IFNs. These cytokines induce an antiviral state in virus infected and neighbouring cells through autocrine or paracrine mechanisms [8]. The type 2 IFN, IFN-γ is produced by T lymphocytes, natural killer cells and plasmacytoid dendritic cells (DC) [9]. IFN-γ has immunostimulatory as well as immunomodulatory roles in innate and adaptive immunity and like type 1 IFNs is able to inhibit viral replication directly. The recently classified type 3 IFNs comprise IFN-λ1 (IL-29), IFN-λ2 (IL-28A), IFN-λ3 (IL28B) and IFN-λ4 [5,6,10]. Most cell types express IFN-λ1–3 in response to virus infection [11,12]. The IFN-λs are amongst the major IFNs induced in airway epithelial cells and myeloid and plasmacytoid dendritic cells are the most potent produces [13]. Like the type 1 IFNs, type 3 IFNs are induced through sensing of viral infection through pattern recognition receptors [14,15].

**Figure 1:**
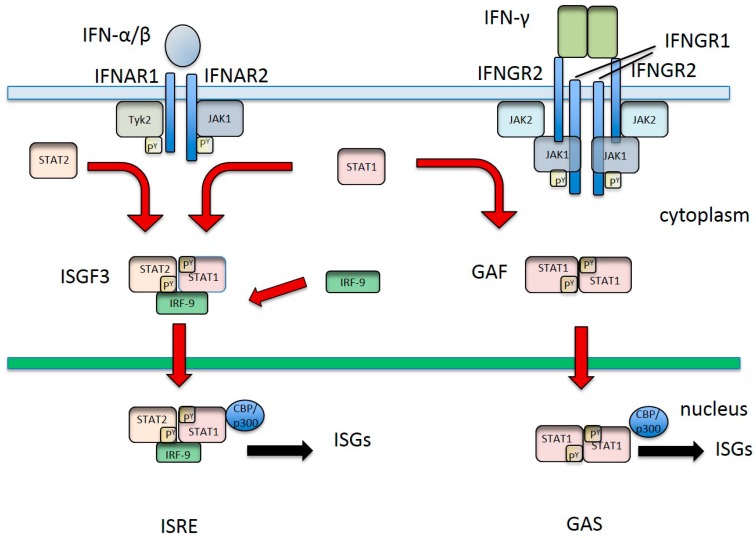
Schematic diagram of the IFN signalling pathways induced by IFN-α/β and IFN-γ (see text for details). Adapted by the author from [29].

IFNs induce the IFN effector response via the JAK/STAT pathway (Figure 1). An excellent review of the non-canonical pathways of type 1 signalling has been published recently [16] and in this review only the canonical pathways will be described. The signal transduction pathways induced by IFN-α/β and IFN-λ are essentially the same but use different receptors. Type 1 IFNs signal through a heterodimeric transmembrane receptor that comprises the subunits IFNAR1 and IFNAR2. The type 3 IFNs signal through the receptor IL-28R that comprises IL-10R2 (also called CRF-2) and IL28RA (also called IFNLR1, CRF2–12) [17]. IFN-γ signals through a heterodimeric transmembrane receptor that consists of the subunits IFNGR1 and IFNGR2. Type 1 IFN engagement with the receptor results in the dimerisation of the IFNAR1 and IFNRA2 chains of the receptor and auto-phosphorylation of the receptor associated kinases JAK1 and tyrosine kinase 2 (Tyk2). In a similar fashion, IFN-λ receptor binding leads to dimerisation of the IL-10R2 and IL-28RA chains and auto-phosphorylation of the receptor associated kinases JAK1 and Tyk2 [13]. The JAKs phosphorylate the intracellular domains of the receptors to establish docking sites for STAT1 and STAT2. STAT1 and STAT2 are subsequently phosphorylated by the JAKs [18]. Phosphorylated STAT1 (Tyr^701^) and STAT2 (Tyr^690^) form a heterodimer that binds IFN regulatory factor IRF9 (also known as p48 or ISGF3 γ) in the cytoplasm forming the heterotrimeric transcriptional factor complex IFN-stimulated gene factor 3 (ISGF3). ISGF3 translocates to the nucleus where it binds the *cis* element known as IFN-stimulated response element (ISRE) [19]. STAT1 and IRF9 make precise contact with the ISRE element while STAT2 makes general contact [19]. This binding step drives the transcription of hundreds of ISGs such as double-stranded RNA-dependent protein kinase R (PKR), 2′,5′-oligoadenylate synthetase (OAS) and myxovirus resistance 1 (Mx1) that are involved in the anti-viral state [1]. IFN-γ engagement with IFNGR1 and IFNGR2 induces dimerisation of the chains that results in autophosphorylation of the receptor associated kinases JAK1 and JAK2. Although the canonical pathway of JAK/STAT signalling mediated by IFN-γ is often reported to involve only the phosphorylation of the transcriptional factor STAT1 [20], a role of STAT2 in IFN-γ signalling has been reported by a number of investigators [21,22,23]. The phosphorylated STAT1 molecules homodimerise to form γ activation factor (GAF) that translocates to the nucleus where it binds to IFN-γ responsive cis element γ activation sequence (GAS), thereby inducing the upregulation of ISGs. In addition, the Type 1 IFN signalling pathway is also mediated through the p38 mitogen activated protein (MAP) kinase pathway [24]. Both the type 1 IFN response and the IFN-γ response leads to the upregulation of hundreds of effector molecules, many of which inhibit and kill viruses and most of these effectors are stimulated by both responses [25,26,27,28]. 

A major threat to viruses are the type 1 and type 3 IFNs, since they are produced in abundance from virus infected cells and induce a rapid anti-viral response in neighbouring cells in the vicinity of the infection. The literature mostly describes viruses that target the JAK/STAT response induced by type 1 IFNs rather than the type 2 IFNs although many viruses have the ability to modulate both pathways in vitro where the pathways have factors in common such as STAT1. Whether viruses in fact target both pathways in vivo depends on the cells and tissue they infect. As described above, the JAK/STAT pathway that is induced by IFNs is a relatively uncomplicated primordial pathway and the factors encoded involve just a few non-redundant genes. It is perhaps this uncomplicated nature of the JAK/STAT pathway that leaves the host vulnerable to virus manipulation. The mechanisms that viruses use to target the JAK/STAT pathway are numerous. For example, STAT1 and STAT2 are targets for the majority of viruses that manipulate the JAK/STAT pathway and the mechanisms involve ubiquitination and degradation and dephosphorylation. The STATS may be sequestered, delocalised and blocked from phosphorylation by JAKs. In some cases, the virus induces the upregulation of suppressor of cytokine signalling cellular genes (SOCS) that regulate the pathways in question. In addition, many viruses target more than one factor in the IFN signalling pathway and there are many examples in the paramyxoviruses (reviewed in [30]). Respiratory syncytial virus targets both IFN-α/β and IFN-γ mediated transcriptional activation by two distinct mechanisms [31]. All vector-borne flaviviruses studied to date suppress host innate immune responses to infection by inhibiting type-1 mediated JAK/STAT signal transduction [32]. The virulence of a specific strain can be correlated with its susceptibility to the antiviral effects of IFN-α/β [33]. The ability to manipulate the JAK/STAT pathway is also associated with virulence and pathogenesis in virulent and non-virulent strains [34]. In addition, encoding multiple IFN antagonists correlates with high virulence and in the case of some flaviviruses contributes to their broad host range by overcoming the IFN response in multiple species [32]. It has become increasingly clear that the modulation of the JAK/STAT pathway is critical for those viruses that establish chronic or persistent infections but many viruses that cause acute infections such as poxviruses also target the JAK/STAT pathway.

It should be noted that viruses also modulate the IFN response in other ways. Many viruses, in particular large DNA viruses such as poxviruses and herpes viruses, encode numerous factors that target Toll-like receptors and RIG-I-like receptors that are considered to be the main receptors for type 1 IFN induction, and nucleotide-oligomerisation domain-like receptors [30,35,36]. In addition, viruses also target IFN effectors such as PKR and OAS [36]. In this review only viral proteins that modulate the JAK/STAT signalling pathway are reviewed. The barrier presented to viral replication from activation of the JAK/STAT pathway by type 1 and type 3 IFNs is formidable and it is likely that all mammalian viruses have evolved genes to combat its effects. Many viruses target the IFN signalling pathway at multiple stages within the cascade, again illustrating the importance of this pathway in combating viral replication. Given our current understanding of the JAK/STAT pathway induced by type 1 and type 3 IFNs, examples can now be found of virus manipulation of all components of the pathway with the exception of the type 3 IFN-receptor. This review illustrates the extraordinary diversity of strategies that viruses have evolved to do this and the implications of these findings for the design of new anti-viral drugs and live attenuated vaccines.

## 2. Targeting the IFN Receptor

Several viruses block IFN-α/β and IFN-γ induced signalling by disrupting the function of the IFN receptor by either forming complexes with receptor subunits, reducing receptor expression or by inducing the degradation of receptors. In addition, poxviruses produce secreted IFN-receptor-like decoy factors and herpes simplex virus 1 induces the secretion of an IFN type 1 antagonising protein.

Measles virus specifically suppresses the IFN-α induced antiviral state but not IFN-γ induced signalling [37]. Viral inhibition of JAK1 phosphorylation by IFN-α suggested that the virus was targeting the signalling pathway upstream of JAK at the level of the type 1 IFN receptor. It was subsequently shown that the IFNAR1 chain forms a complex with the measles virus accessory proteins C and V, and the receptor scaffold protein RACK1 and STAT1 [37]. Moreover the composition of this complex did not change with IFN-α treatment of infected cells. It is thought that the functional disorder of the IFN receptor complex is due to freezing of the receptor through the association with C and V proteins of measles virus [37].

Influenza A viruses disrupt JAK/STAT signalling in part by reducing the expression of the IFN receptor. The multifunctional non-structural protein 1 (NS1) encoded by influenza A viruses predominantly targets IFN by reducing its production [38] but in addition also has the ability to inhibit IFN signalling [39]. NS1 targets both chains of the IFNAR receptor by reducing their expression at the transcriptional level.

The herpes virus Epstein Barr virus encodes the protein BZLF1 that decreases the ability of IFN-γ to activate a number of genes including IRF-1, p48 (IRF9), and MHC class II expression, however it also inhibits phosphorylation of STAT1 and decreases the expression of the IFN-γ receptor [40]. In addition, Epstein-Barr virus targets the IFN receptor by degradation. The latent membrane proteins LMP2A and LMP2B modulate signalling from type 1 and type 2 IFN receptors, not by affecting cell surface levels of IFNRs, but by inducing an accelerated turnover through a process requiring endosome acidification [41].

### Targeting IFN Receptor Binding

A strategy that a small number of viruses have evolved to counteract the biological activity of IFNs is to intercept these cytokines before they bind to their cognate receptors. In general the viral encoded molecules mimic the extracellular binding domain of host cytokine receptors and are secreted from virus-infected cells. Poxviruses, with their large genomes, appear to have captured genes from their host species by unknown mechanisms. Myxoma virus is a highly infectious disease of rabbits that causes swelling of the mucous membranes and inflammation and is usually fatal. Myxoma virus encodes a protein, T7, that is an IFN-γ like receptor and specifically binds rabbit IFN-γ and was the first example of this strategy used against the interferon family [42,43]. Moreover, the deletion of the *M-T7* gene from the myxoma virus genome showed that it was a critical virulence factor for the development of myxomatosis in rabbits [43]. IFN-γ receptors are also highly conserved in all orthopoxviruses including variola (the causative agent of smallpox), vaccinia virus, cowpox virus, camelpox virus and ectromelia virus [44,45]. In addition, the avian poxvirus fowlpoxvirus has also been shown to encode an IFN-γ binding protein but unlike its mammalian counterparts exhibits no significant homology to any known viral or cellular proteins [46].

A number of orthopoxviruses also express a type I IFN binding protein. Vaccinia virus encodes a secreted protein B18R that has significant regions of homology with the alpha subunits of the mouse, human and bovine type I IFN receptors [47]. B18R has three immunoglobulin domains that function as a soluble receptor for IFN-α/β [48]. The secreted IFN-α/β receptors not only bind soluble cytokine but also bind to infected and uninfected cells to protect them from the antiviral effects of IFN-α/β [48].

Herpes Simplex Virus 1 has evolved a mechanism similar to that of orthopoxviruses to inhibit the type I IFN response [49]. The expression of the HSV-1 encoded ICP27 protein in virus-infected cells induces the secretion of an IFN type I antagonising protein. Similar to orthopoxviruses, the mechanism that HSV-1 has evolved not only inhibits IFN signalling in infected cells but also in neighbouring cells to allow the virus to spread.

## 3. Targeting Tyk2 and JAK1 IFN Receptor Associated Kinases

The Janus kinase family includes JAK1, JAK2, JAK3 and Tyrosine kinase (Tyk2). Tyk2 and JAK1 are major targets of both RNA and DNA viruses.

Flaviviruses are transmitted to humans by mosquitoes and ticks and cause millions of infections annually. West Nile virus, Japanese encephalitis virus, dengue virus, yellow fever virus and tick-borne encephalitis virus are associated with morbidity and mortality worldwide. All flaviviruses studied to date inhibit host innate responses to infection by inhibiting type 1 IFN mediated JAK/STAT signal transduction and there are a number of viral encoded factors involved [50,51,52,53,54]. The flavivirus genome encodes one large polyprotein that is cleaved into three structural proteins and seven non-structural proteins [32]. NS2B, NS4A and NS5 have all been shown to prevent STAT1 activation to various degrees. The multifunctional factor NS5 has been identified as a major determinant of virulence and is highly conserved among flaviviruses due to the fact that it also encodes the viral methyl transferase and RNA dependant RNA polymerase. The block in JAK/STAT signalling for the above flaviviruses is a result of targeting JAK phosphorylation. In addition, dengue virus also targets STAT2 by degradation [55]. The ability of the flaviviruses to inhibit JAK/STAT signalling may explain the limited success of IFN-α2a as a therapeutic for flavivirus infections in human trials [56,57].

Respiratory syncytial virus (subfamily *Pneumovirinae*), that is the causative agent of lower respiratory tract infections, bronchiolitis and pneumonia during (early) childhood impairs both IFN-α/β and IFN-γ signalling in primary murine alveolar macrophages and in human macrophages (THP-1 cells) by two distinct mechanisms [31]. Respiratory syncytial virus impairs IFN-β signalling through a mechanism that involves Tyk2 phosphorylation and by modulating the STAT1-CBP protein–protein interactions and transcriptional complex assembly (see below Targeting IRF9 and ISGF3 cofactors). It is thought that a phosphatase-mediated mechanism is involved in reducing phosphorylation of Tyk2.

The E6 protein expressed by the malignant strain of the human papilloma virus-18 physically associates with Tyk2 within domains JH6–JH7 that are critical for binding of Tyk2 to IFNAR1 resulting in decreased phosphorylation of Tyk2 [58]. Similarly, Kaposi’s sarcoma-associated herpes virus blocks the type 1 IFN response by expressing the protein RIF (regulator of IFN function) that forms a complex with IFNAR subunits JAK1 and Tyk2 and the STAT2 transcription factor [59]. The formation of the complexes leads to inhibition of the activation of JAK1 and Tyk2 and abnormal recruitment of STAT2 to the IFNAR1 subunit. Likewise, myxoma virus modulates type 1 IFN signalling by blocking the phosphorylation of Tyk2 specifically and is the only poxvirus known to use this mechanism [60]. The paramyxovirus, measles virus blocks STAT1 and STAT2 phosphorylation by interaction of its V protein (N-terminal domain) with JAK1 [61,62].

Hepatitis C and B viruses cause chronic infections and use a unique strategy to impair IFN-α signalling [63]. Hepatitis B and C are known to up-regulate the expression of a major serine/threonine phosphatase 2A (PP2A) that is ubiquitously expressed in all cell types. PP2A associates with JAK1/Tyk2 and STAT1 and reduces JAK1/Tyk2/STAT1 phosphorylation resulting in an impairment of the IFN-α-induced response. Although PP2A is not required to block the effects of IFN-α, it is required for viral replication and could play a role in the establishment of chronic infection.

Several viruses are known to disrupt JAK1 and Tyk2 at the mRNA level. Adenoviruses are DNA viruses that cause a broad spectrum of infections but some serotypes can cause severe respiratory disease. The specific effects of adenovirus type 5 have been studied in human tracheobronchial epithelial cells. The virus was shown to inhibit type 1 IFN signalling by decreasing JAK1 mRNA levels [64]. Blue tongue virus, a segmented double stranded RNA virus, causes a severe disease in ruminants. The inhibition of the type 1 IFN response by blue tongue virus involves two different mechanisms [65]. During early infection (within 24 h in cell culture), the virus blocks the phosphorylation and nuclear translocation of STAT1; however, during late infection, the virus modulates the IFN-1 response by inducing the downregulation of JAK1 and Tyk2 protein expression [65].

Human cytomegalovirus has evolved several mechanisms to disrupt JAK/STAT signalling. One of these mechanisms involves reducing JAK1 levels by degradation via the proteasome complex [66,67]. Miller et al. [66] have demonstrated that human cytomegalovirus inhibits the expression of MHC class I, IFN regulatory factor-1, MxA and 2′,5′-oligoadenylate synthetase gene expression in fibroblasts and endothelial cells stimulated with IFN-α by decreasing the expression of JAK1 and IRF-9. In addition, they have shown that human cytomegalovirus inhibits MHC II expression in IFN-γ stimulated human endothelial cells and fibroblasts and this effect is also associated with a marked decrease in JAK1 [67].

### Targeting JAKs by Viral Induction of Suppressor of Cytokine Signalling (SOCS) Factors

In recent years, a number of viruses have been shown to block JAK/STAT signalling by upregulating members of the suppressor of cytokine signalling (SOCS) proteins SOCS1 and SOCS3. SOCS are normally induced by cytokine stimulation and they serve to interfere with signalling, not only from the inducing cytokine in a classic “negative-feedback” loop, but also regulate signalling downstream of other cytokines [68]. All SOCS proteins contain an SH2 domain and a C-terminal SOCS box domain that is critical for proteasomal degradation of SOCS-associated proteins. Furthermore, SOCS1 and SOCS3 contain an additional kinase inhibitory region (KIR) that is essential for inhibition of kinase activity. SOCS1 and SOCS3 proteins directly bind to JAKs through the SH2 domain and regulate their function by proteasomal degradation or by inhibiting their kinase activity.

The first report that demonstrated a viral protein was capable of inducing the expression of SOCSs molecules came from a study of hepatitis C virus. Hepatitis C is a major cause of chronic hepatitis, end-stage cirrhosis and hepatocellular carcinoma. Eighty per cent of patients newly infected with hepatitis C virus develop chronic infection and treatment with IFN-α, has only achieved moderate results [69]. Studies have shown that the hepatitis C virus core protein, whose main function is to form the capsid shell, may be part of the molecular basis of IFN-α unresponsiveness [70]. Bode et al. [70] showed that overexpression of the hepatitis C virus core protein in hepatic cells inhibits IFN-α induced activation of STAT1 and was associated with the induction of SOCS3-mRNA expression. Their findings suggest that the hepatitis C virus core protein impairs IFN-α induced signal transduction by inducing SOCS3 expression and furthermore it was able to rescue influenza A virus lacking its own IFN-antagonist. Since its discovery several other viruses have also been reported to modulate the JAK/STAT pathway by upregulating SOCS3. Varicella zoster virus that causes varicella (chickenpox) and herpes zoster (shingles) induces increased levels of SOCS3 [71]. In addition influenza A virus inhibits type 1 IFN signalling through the induction of SOCS3 expression [72].

Other viruses have been shown to modulate the JAK/STAT pathway by upregulating SOCS1. Herpes simplex virus 1 is a large double-stranded DNA that infects epithelial cells and establishes a latent infection in the sensory ganglia that can become reactivated [73]. Herpes simplex virus 1 infects 60%–80% of people worldwide and persists for life. Keratinocytes are important for the acute phase of infection with subsequent persistence in sensory nervous tissue. Infection is characterised by a strong cytokine response in infected cells with the induction of large amounts of type 1 IFNs and pro-inflammatory cytokines [74]. Herpes simplex virus 1 has evolved multiple strategies to inhibit the effects of IFN [75,76,77,78] and one of these mechanisms involves upregulating SOCS1 [79]. Rhinovirus infections can cause asthma and induction of type 1 IFNs and IFN-γ is impaired in patients. Gielen et al. [80] found that SOCS-1 expression in tissues from asthmatic patients was related to asthma severity. Furthermore, respiratory syncytial virus has also been shown to upregulate the SOCS members SOCS1, SOCS3 and CIS in HEP-2 cells [81], suggesting the involvement of SOCSs in respiratory virus infections.

A viral encoded SOCS has been reported for a virus that infects fish but no such genes have been reported for mammalian viruses [82]. Infectious spleen and kidney necrosis virus belongs to the family *Iridoviridae* and was isolated from mandarin fish in which the virus is highly infectious and pathogenic. Sequence and bioinformatic analyses of the virus genome demonstrated that open reading frame 103R encodes a predicted viral SOCS with high homology to SOCS1 but lacks a SOCS box domain. The novel viral SOCS protein was shown to interact with the JAK1 protein and to inhibit tyrosine kinase activity in vitro [82]. Of evolutionary significance is the fact that it inhibited the IFN-α-induced JAK/STAT signal transduction pathway in human liver cancer (HepG2) cells [82], illustrating the remarkable conservation of the JAK/STAT pathway across the animal kingdom.

## 4. Targeting STAT1 and STAT2

The majority of viruses that impair the JAK/STAT signalling pathway have evolved mechanisms to target STAT1 and STAT2. The mechanisms used are diverse and include viral phosphatases, retention of suppression of STATS in the cytoplasm, inhibition of STAT phosphorylation, degradation of STATS through the proteasome and blocking of nuclear import.

### 4.1. Blocking Phosphorylation of STATs

A number of viruses inhibit phosphorylation of STAT1 or STAT2 or both either directly or indirectly as described above by interaction with JAKs or altering the levels of JAKs. In general, this requires new viral gene expression. Phosphorylation of STAT1 at Tyr^701^ is critical for activation and viruses have evolved proteins to specifically block this process. Blocking STAT1 phosphorylation prevents either homodimerisation in the case of IFN-γ induced signalling to form GAF, or the formation of a trimeric complex of ISGF3 with STAT2 and IRF9 in IFN-α/β induced signalling. STAT1 may be a common target for some viruses since it is required for both type 1 IFN as well as IFN-γ induced signalling.

The paramyxovirus family includes the human viruses measles virus, mumps virus, parainfluenza viruses, Hendra virus and Nipah virus. Hendra virus and Nipah virus are zoonoses and cause up to 70% mortality in humans. The paramyxoviruses disrupt STAT signalling by various mechanisms (reviewed in [29,30]) including direct binding to STAT1 and STAT2 to prevent phosphorylation by way of its accessory factors. The paramyxoviruses are small RNA viruses that contain only six genes to express essential factors for structure and replication. However they express a number of accessory protein isoforms within the *P* gene (phosphoprotein) by RNA editing that results in frame-shifts [30]. Up to nine proteins are encoded within the *P* gene including V, C and P and a protein named W, D or I. The V proteins are generally considered to be the principle IFN antagonists, however, the P, W and C proteins also play diverse roles in antagonising IFN signalling as described below. The V protein has multiple functions in disrupting signalling, including direct binding to STAT1 and STAT2 to prevent phosphorylation by way of their N-terminal and C-terminal regions. This mechanism is found for mumps virus, Hendra virus, Nipah virus and the swine virus Rinderpest virus. Rinderpest virus and Nipah virus also block STAT1 phosphorylation by way of their W and P proteins, respectively. In addition Sendai virus inhibits IFN-induced tyrosine phosphorylation of STATs by its C proteins [29,83,84,85]. All four C proteins bind STAT1 but the largest C protein also ubiquitinates STAT1 and induces its degradation [84]. Moreover, deletion of the *C* gene abolishes the ability to block the IFN-α/β response [29,85]. In a more recent report, the C protein has been shown to form high molecular weight complexes with STAT1 [86] (see below, 4.3).

Epstein–Barr virus is a ubiquitous γ-herpes virus that causes infectious mononucleosis [87]. The virus infects epithelial cells and converts to a latent state in B cells. Epstein–Barr virus encodes the proteins BZLF1 and BRLF1 that induce the lytic cascade of gene expression and BZLF1 also has a role in inhibiting the IFN response. Curiously, it inhibits IFN-γ induced STAT1 tyrosine phosphorylation and nuclear translocation but not IFN-α induced STAT1 tyrosine phosphorylation. In addition, it reduces JAK1 and JAK2 tyrosine phosphorylation and decreases the expression of IFNGRA [40]. The immediate-early gene ICP27 of herpes simplex virus 1 has been identified as playing a critical role in down-regulating STAT1 phosphorylation by some unknown mechanism and preventing the accumulation of STAT1 in the nucleus [75].

The α-RNA virus Chikungunya virus is an emerging human pathogen transmitted by the *Aedes* mosquito species and is present in the Indian Ocean region [88]. Chikungunya virus is resistant to IFN once RNA replication has been established and efficiently blocks STAT1 phosphorylation and/or nuclear translocation in cells induced by either type 1 or type 2 IFNs. The region of the genome responsible for blocking IFN signalling has been mapped to the *NSP2* gene. The NSP2 protein functions as a protease and helicase but has also been identified as a potent inhibitor of IFN-induced JAK/STAT signalling. Studies in a close relative, Sindbis virus, have shown that mutation of proline to serine at position 762 in a conserved region of NSP2 reduced cytopathicity [89]. From these findings Fros et al. [88] were able to demonstrate that a proline to serine mutation in NSP2 of Chikungunya virus within the same conserved region (position 718) reduced its ability to inhibit IFN signalling and productive infection [88]. The exact mechanism by which this protein inhibits JAK/STAT signalling has not been established but it does not involve degradation of STATs.

Respiratory syncytial virus uses different mechanisms to inhibit the type 1 IFN response in epithelial cells and dendritic cells (also see above inhibition of Tyk2 phosphorylation in macrophages and targeting ISGF3 assembly (Section 5) [31]). Dendritic cells act as a portal for virus invasion and are present in the respiratory tract airway epithelium in high numbers. In epithelial cells this virus degrades STATs and in dendritic cells it inhibits STAT1 and STAT2 phosphorylation and nuclear translocation [90].

Marburg virus is a member of the filovirus family of negative strand RNA viruses and causes highly lethal haemorrhagic fever in humans and in non-human primates. Marburg virus encodes a matrix protein VP40 that inhibits phosphorylation of STAT1 and STAT2 [91].

### 4.2. Dephosphorylation of STATS: Viral and Cellular Induced Phosphatases

A small number of viruses use a strategy to inhibit IFN signalling by dephosphorylating activated STATs. Poxviruses encode a phosphatase that has roles in morphogenesis, as well as dephosphorylating STATs while other viruses induce cellular phosphatases to inhibit IFN signalling.

The orthopoxvirus, vaccinia virus, blocks IFN-γ signal transduction by a phosphatase (VH1) that is incorporated into the viral particle and is released upon entry of the virus into cells and uncoating of viral cores [92]. Significantly, virions containing reduced levels of VH1 fail to block the IFN-γ signalling pathway suggesting that this is the only mechanism used to block signalling in addition to the secreted IFN receptor homologues [44,45,47,48]. The vaccinia virus phosphatase has dual specific activity and dephosphorylates STAT1 at Tyr^701^ as well as Ser^727^ when the signalling pathway is activated by IFN-γ [93]. Since its discovery, VH1 has also been shown to dephosphorylate STAT1 and STAT2 induced by type 1 IFNs [94]. VH1-like phosphatases have also been demonstrated for a number of other poxviruses including the highly virulent variola virus (Smallpox) [95,96]. The parapoxvirus orf virus, which induces pustular skin lesions in sheep and goats and can be transmitted to humans [97], has the ability to modulate the JAK/STAT pathway by inhibiting signalling by both type 1 and type 2 IFNs [98]. Interestingly, although orf virus encodes a protein with homology to VH1, the virus only dephosphorylates IFN-γ activated STAT1 at Tyr^701^ and not Ser^727^ in human cells, suggesting a possible evolutionary divergence of this virion associated enzyme. The viral phosphatase is a novel mechanism and has not been found in other viruses outside the *Poxviridae* family.

Human cytomegalovirus is a ubiquitous β-herpesvirus that can persist in the host by its ability to enter latency [99]. Human cytomegalovirus has evolved several mechanisms to escape the host immune response [66,100]. One of these mechanisms involves inhibition of IFN-γ-induced STAT1 tyrosine phosphorylation by activating a cellular phosphatase and is dependent on human cytomegalovirus transcription [100]. Src homology region 2 domain-containing phosphatase 2 (SHP2) is a ubiquitous phosphatase involved in the regulation of IFN-γ-mediated tyrosine phosphorylation of activated STAT1. Several mechanisms have been identified by which human cytomegalovirus inhibits IFN-γ signalling but one of these mechanisms appears to involve the activation of SHP2 that is responsible for the down-regulation of IFN-γ induced STAT1 phosphorylation. Similarly, a dual specificity phosphatase 1 has been reported to be specifically upregulated in the liver of patients with chronic hepatitis C virus that do not respond to treatment with peginterferon [101]. Silencing of the cellular phosphatase in hepatoma cells stably expressing the hepatitis C virus replicon, enhances activated STAT1 and ISG expression suggesting a potential target to treat hepatitis C infection [101].

### 4.3. Sequestration of STATs in High Molecular Weight Complexes

Several members of the *Paramyxoviridae* family, including Nipah virus and Sendai virus, evade IFN-α and IFN-γ induced intracellular responses by preventing STAT activation and nuclear accumulation by forming high molecular weight complexes [86,102]. The Nipah virus encoded V protein accumulates in the cytoplasm by a Crm1-dependent mechanism and associates tightly with both STAT1 and STAT2. As a result, the proteins are retained in the cytoplasm preventing both IFN-induced STAT activation and nuclear translocation. Sendai virus also uses a similar mechanism. The Sendai virus encoded C protein inhibits both IFN-α/β and IFN-γ by binding the N-terminal domain of STAT1 [86]. In Sendai virus infected cells, the phosphorylation of STAT1 at Tyr^701^ is enhanced, although STAT1 does not form an active transcriptional complex. The C protein blocks signalling by interfering with the domain arrangement of the STAT1 dimer, leading to the accumulation of phosphorylated STAT1 in the cytoplasm and the formation of high molecular weight complexes.

### 4.4. Decreasing the Basal Levels of STATs by Degradation and Transcriptional Suppression

Members of the *Paramyxoviridae* family, adenovirus, human cytomegalovirus, dengue virus and Zika virus, have evolved specific proteins that directly suppress IFN signalling by reducing the concentration of cellular STAT proteins [29,55,103,104,105].

Almost all members of *Rubulavirus* genus of the subfamily *Paramyxovirinae* have acquired the ability to specifically target either STAT1 or STAT2 for degradation using their accessory V protein encoded by the *P* gene [29,103,106,107,108,109]. Parainfluenza virus 5 (previously simian virus 5) and mumps virus reduce the levels of STAT1 by proteasome-mediated degradation. The parainfluenza virus 5 V-protein is a structural protein associated with nucleocapsids of the virion [110] and degrades both phosphorylated and non-phosphorylated forms of STAT1 [109]. Human parainfluenza 2 also has the ability to reduce STAT levels but unlike Simian virus and mumps virus, it induces the degradation of STAT2 but not STAT1 [108]. In addition the Newcastle disease virus from the *Avulavirus* genus of the subfamily *Paramyxovirus* targets STAT1 for degradation [111].

Respiratory syncytial virus has evolved several strategies to inhibit IFN-α/β signalling that involve inhibiting Tyk2 phosphorylation (refer to Section 3) and modulating STAT1-CBP protein–protein interactions and transcriptional complex assembly [31,105] (refer to Section 5). In addition respiratory syncytial virus induces ubiquitination of STAT2 by an elongin-cullin E3 ligase and degradation via the proteasome [105,112,113].

The recalcitrance of adenovirus to IFN is largely mediated through the E1A proteins and can be partly attributed to the lowering of the levels of trans-activating factors required for both IFN-γ and IFN-α/β signalling [104]. E1A proteins are expressed early in adenovirus-infected cells and are potent regulators of gene expression and cell function. In E1A-expressing HeLa cells, IFN-γ signalling is blocked by a reduction in the levels of the STAT1α isoform protein and IFN-α signalling is blocked by a reduction in the level of p48 (IRF9).

Dengue virus achieves high titres in humans despite the induction of high levels of IFN-α. In addition IFN-α/β has little effect on virus replication in cell culture after replication has been established. Dengue virus has evolved several mechanisms to inhibit IFN signalling and one these mechanisms involves the down-regulation of STAT2 [55]. It has recently been shown that the non-structural protein NS5 of the flavivirus Zika virus targets STAT2 for degradation via the proteasome pathway [114] and specific strains of human cytomegalovirus also induce the degradation of STAT2 through the proteasome pathway [115].

### 4.5. Prevention of Nuclear Accumulation/Import of Activated STAT1 

Several viruses encode proteins that inhibit IFN signalling by preventing IFN-induced STAT1 nuclear accumulation. Rabies virus, a member of the *Rhabdovirus* family, antagonizes both type 1 and type 2 IFN signalling. The rabies virus P protein is a cofactor of RNA polymerase but also binds the DNA-binding domain and the coiled-coil domain of STAT1 [116]. The multifunctional P protein also inhibits both IFN-α and IFN-γ induced transcriptional responses by a mechanism that does not interfere with STAT1 phosphorylation but impairs nuclear translocation of STAT1. The *Paramyxovirus* mapuera virus uses a similar mechanism to block signalling where its V protein binds to both STAT1 and STAT2 to prevent nuclear accumulation [117] and the mumps virus NP protein co-localises with STAT2 in punctate aggregates in the cytoplasm [118].

Nuclear transport of phosphorylated tyrosine STAT1 occurs via a subset of karyopherin alpha nuclear transporters. Ebola virus is a member of the filovirus family and antagonizes STAT1 signalling by encoding the minor matrix protein VP24 that binds a unique nuclear localisation site on karyopherin-alpha 5 to inhibit STAT1 nuclear import [119].

## 5. Targeting IRF9 and ISGF3 Transcriptional Cofactors

A number of viruses block IFN-α/β signalling by targeting either IRF9 that is required for the formation of the transcriptional trimeric complex ISGF3 or transcriptional co-activators of ISGF3, CBP and p300. This strategy is found among viruses that cause persistent infections and oncogenesis including human papilloma virus, human cytomegelovirus, varicella zoster virus and human T-cell leukemia virus.

Human papilloma viruses are small dsDNA viruses that cause skin and anogenital warts and cervical cancer [120]. The discovery of a human papilloma virus encoded factor that blocks the formation of the ISGF3 complex may go someway towards explaining the lack of responsiveness to IFN treatment [121]. Expression of the multifunctional oncoprotein E7 has been shown to inhibit the IFN-α response by disrupting the formation of the ISGF3 complex by binding IRF9 thus inhibiting the translocation of IRF9 to the nucleus in IFN-α stimulated cells [121]. The human cytomegalovirus causes extensive morbidity and mortality in neonatal and immunocompromised patients. Human cytomegalovirus infection of human embryonic lung fibroblasts showed that the virus specifically reduces the levels of IRF9 and JAK1, resulting in multiple lesions within the signalling pathway [66]. This novel immune escape mechanism is thought to be a major means by which human cytomegalovirus establishes persistence. Porcine bocavirus is a newly emergent parvovirus and shows clinically high co-infection prevalence with other pathogens in post-weaning multi-systemic wasting syndrome and diarrheic piglets [122]. Screening of structural and non-structural proteins found that the non-structural protein NS1 inhibited IFN-β production and antagonized IFN signalling by reducing the DNA-binding activity of ISGF3 [123]. NSP1 was shown to interact specifically with the DNA-binding domain of IFR9. Varicella-zoster virus establishes latency in the sensory ganglion and has evolved several mechanisms to inhibit type 1 IFN signalling [124]. One of the mechanisms involves a protein encoded by *ORF63* that induces the degradation of IRF9 and indirectly blocks STAT2 phosphorylation. In addition hepatitis C virus has been reported to inhibit the binding of ISGF3 to the ISRE element by its core protein [125].

Several viruses target the transcriptional co-activators of ISGF3 CBP and p300. These include the oncogenic retrovirus, human T-cell leukaemia virus type 1 that causes adult T-cell leukaemia/lymphoma [126]. Human T-cell leukaemia virus type 1 associated oncogenesis is mostly due to the expression of the viral regulatory protein Tax [127]. The Tax protein has multiple roles in transcriptional activation including a role as an IFN-α antagonist that counteracts the function of the ISGFR3 transcriptional complex. Tax has been shown to interfere with JAK/STAT signal transduction by interacting with CBP/p300 in competition with STAT2, thereby inhibiting the transcriptional activation of the STAT2-containing ISGF3 complex [128]. Respiratory syncytial virus inhibits IFN-γ and IFN-α/β in macrophages by employing distinct mechanisms. In addition to the mechanisms described above by which it blocks IFN-β signalling, respiratory syncytial virus blocks IFN-γ induced signalling by disrupting the assembly of the transcriptional complex [31]. STAT1 exists naturally as both STAT1α and STAT1β isoforms through alternative splicing. In respiratory syncytial virus infected macrophages, STAT1β has a dominant negative function due to its inability to interact with the co-activator CBP/p300, thus inhibiting IFN-γ transcriptional activation in macrophages [31].

## 6. Targeting the JAK/STAT Pathway by Viral Host Shut-Off

Herpes simplex viruses type 1 and 2 suppress host cell protein synthesis by a phenomenon known as virion associated host shuttoff (VHS) [129]. VHS is mediated by a component of infecting virions that is expressed by the gene *UL41* [130]. UL41 is a ribonuclease that was initially shown to disrupt polysomes and induce the rapid degradation of host and viral mRNAs [130,131]. Since its discovery, VHS has been linked to occlusion of the IFN JAK/STAT signalling pathway [76,132]. Herpes simplex virus 2 VHS-deficient mutants are highly attenuated in mice*.* In contrast the replication of this mutant is largely restored in mice lacking the IFN-α/β receptor. In addition, infection studies in vitro have shown that the growth of the VHS-deficient mutant was similar to that of wt virus in MEF cells lacking the IFN-α/β receptor. This study demonstrated that VHS counteracts the type 1 IFN response by inhibiting the activation of the anti-viral state by a mechanism that involves the degradation of type 1 IFN and ISG mRNAs [132]. Similar studies performed with a herpes simplex virus 1 mutant lacking the *UL41* gene, has shown that the disappearance of JAK1 and STAT2 in virus-infected cells is related in part to degradation of cellular mRNAs [76].

## 7. Targeting the JAK/STAT Pathway by Upregulating Cellular MicroRNA

MicroRNA (miRNA) has recently been implicated in viral immune evasion. Hepatitis C virus infection is one of the main causes of liver disease and a prophylactic anti-hepatitis C virus vaccine is unavailable. In chronic hepatitis C virus infection both the innate and adaptive immune signalling mechanisms are adversely affected [133]. The miRNAs have been associated with the control of a number of biological processes, including IFN signalling. Hepatitis C virus infection is known to alter the miRNA expression profile of host cells and several miRNAs are known to participate in innate and adaptive responses to hepatitis C virus infection [134,135,136,137,138]. During infection of human heptocytes, miRNA-373 is upregulated. The increased level of miRNA-373 in the cell suppresses JAK1 and IRF9 expression and suppresses responsiveness to type 1 IFN [139]. Furthermore it was shown that knock-down of miRNA-373 restricts hepatitis C virus replication.

## 8. Development of Live Attenuated Vaccines by Mutation of Viral-Encoded IFN-Antagonists

The critical role of the IFN induced JAK/STAT signalling pathway in anti-viral immunity, is highlighted by the ever-growing number of viruses that have evolved strategies to combat its induction. Moreover, such studies have shown a strong correlation between the ability of a virus to inhibit IFN induced signalling and virulence [34,140]. Other studies using STAT1 knockout mice also highlight the importance of targeting the JAK/STAT1 pathway in virulence and pathogenesis [141,142]. Clearly, the ability of a virus to inhibit IFN signalling is not the only determinant of virulence but it is a critical factor nevertheless. The attenuation of viruses by the mutation of specific genetic elements that encode such factors is being actively pursued for the development of new live attenuated vaccines. Live vaccines are considered more protective than inactivated vaccines [143,144,145,146,147]. Live attenuated vaccines elicit humoral and cellular immunity, whereas traditional killed vaccines act by mainly inducing neutralising antibodies. A number of studies are in their early stages where mutation of viral antagonists is assessed for its effects on virulence and pathogenicity. These include investigations of Nipah virus [102,148,149,150] measles virus [151,152] Sinbus virus [34] and West Nile virus [32]. Other investigations are more advanced and are at the proof-of-concept stage such as studies with recombinant respiratory syncytial virus [153] and influenza virus [154] where human trials have been conducted to assess their vaccine potential.

Members of the genus *Alphavirus*, family *Togaviridae* include a number of human pathogens with a worldwide distribution and are transmitted through mosquito vectors. Sindbis virus, the prototypic alphavirus, causes arthralgia in humans. Infection of mice with Sindbis virus causes encephalomylitis and has provided an excellent model for this viral-induced disease [34,155,156,157]. Two strains of the virus have been used to study a major virulence factor, NSP1 that inhibits JAK/STAT signalling [34,158,159]. The neurovirulent strain AR46 potently inhibits tyrosine phosphorylation of STAT1 and STAT2 in response to IFN-γ and IFN-α/β [34]. In contrast, the closely related Sinbis virus strains Girdwood and TR339 that do no cause detectable disease in adult mice, are relatively inefficient inhibitors of the JAK/STAT signalling pathway. The inhibition of signalling is due to a block in the activation of tyrosine kinases Tyk2 and JAK1, and JAK2 by NSP1 of the neurovirulent strain. Using a panel of AR86/Girdwood chimeric viruses, a single amino acid (threonine at position 538) in NSP1 was shown to be required for virulence and for efficient disruption of STAT1 activation. Moreover changing NSP1 of the Girdwood strain to threonine at position 538 conferred the ability to inhibit STAT1 activation and virulence. Simmons et al. [34] concluded from their studies that NSP1 is a key determinant of virulence in alphaviruses and suggests that the ability to inhibit the JAK/STAT signalling pathway relates to their in vivo virulence potential.

A similar approach to that taken with Sinbis virus has been used to identify a mutation in the NS5 protein of Kunjin virus that results in its attenuation. Kunjin virus is a naturally attenuated subtype of West Nile virus that is endemic in Australia but only rarely causes clinical disease in humans [32]. Molecular genetic studies of Kunjin virus have identified a single amino acid in the major virulence determinant NS5 that likely explains its lack of virulence [32]. By changing a single amino acid residue from serine to phenylalanine at position 653 in NS5, the ability of Kunjin virus to inhibit JAK/STAT signalling was restored. Furthermore, when this amino acid was changed from phenylalanine to serine in the virulent West Nile strain of NS5 it lost its ability to inhibit JAK/STAT signalling. Importantly, the mutation did not disrupt the RNA dependent RNA polymerase and methyl transferase activities of West Nile virus NS5. Studies are underway to examine virulence of these recombinants in animal models [32]. In support of these findings, it has been shown that NS5 of the vaccine strain Japanese encephalitis virus is defective in blocking JAK/STAT signalling [140]. Such studies highlight the importance of NS5 as a virulence factor in the flavivirus family and provide a rational strategy for the construction of live attenuated vaccine strains.

The paramyxovirus measles virus is a leading cause of death among children worldwide. The measles virus P gene encodes three proteins, the phosphoprotein (P) and accessory proteins V and C generated by RNA editing (described above, 4.1). These proteins have roles in replication and transcription and interfere with innate immunity by controlling IFN responses [160,161]. P is a polymerase cofactor and controls IFN signalling [141]. V shares its first 231 amino acids with P [141]. V inhibits IFN signalling [61,162,163] and also inhibits the activity of MDA5 [164]. With a view to attenuating measles virus there has been much interest in mapping the domains and identifying amino acids that control the IFN response. Devaux et al. [141] produced a recombinant measles virus that could no longer antagonise STAT1 function. Amino acids were identified in the V and P proteins that when mutated in the wt virus (STAT1-blind) could not prevent STAT1 translocation into the nucleus in infected cells and resulted in 96 per cent ISRE promoter function. When the STAT1-blind recombinant virus was assessed for virulence in rhesus monkeys, none of the monkeys infected with the STAT1-blind strain developed skin rash, anorexia or diarrhoea seen with wt virus. The recombinant virus could not be detected 14 days post-infection, whereas it could be detected in five of the six monkeys infected with the wt at this time. The findings indicated that measles virus interactions with STAT1 are required to sustain virulence. Moreover the recombinant virus, although unable to control inflammation, produced a humoral and cell mediated response typical of the wt virus suggesting that it may have utility for vaccination.

Respiratory syncytial virus is a leading cause of respiratory tract illness in infants and children worldwide and is a priority for vaccine development [165,166]. The primary strategy for vaccine development has been to construct live attenuated vaccine candidates that can be administered intra-nasally and produce a protective immune response. A number of strategies have been considered to construct a new recombinant vaccine by reverse genetics that include deletion of IFN antagonists [144,153]. Respiratory syncytial virus is a single negative sense RNA that contains 10 genes [144]. Two of these genes encode the non-structural proteins NS1 and NS2 that antagonise the host IFN-α/β system [105,167]. Valarcher et al. [168] showed that deletion of the NS1 and NS2 genes of bovine respiratory syncytial virus increased immunogenicity in calves and studies in chimpanzees have shown that a moderate degree of replication of human respiratory syncytial virus is important to elicit a satisfactory immunogenic response [144]. In a study by Wright et al. [153], three recombinants were constructed that had deletions in the respiratory syncytial virus NS2 gene and of these, two had further deletions in the L gene. The recombinants were assessed in human trials firstly in adults and then children. Although deletion of the NS2 gene further attenuated the virus, none of the recombinant viruses in this study were considered to be satisfactory as a vaccine strain. Nevertheless, the observation that the deletion of the NS2 gene leads to attenuation of respiratory syncytial virus for humans validates the strategy of using reverse genetics for the development of human vaccines.

Influenza viruses cause significant clinical disease annually and historically there has been an increased risk of more serious infection in the elderly [169]. The gradual decline of innate and adaptive responses in the elderly is known as immunosenescence and the trivalent influenza vaccines have been found to be suboptimal [170,171,172,173]. As described above, NS1 encoded by influenza A and B viruses is an antagonist of the IFN response [39]. Influenza A viruses disrupt JAK/STAT signalling in part by reducing the expression of the IFN receptor. NS1 targets both chains of the IFNAR receptor by reducing their expression at the transcriptional level. In addition, NS1 induces the upregulation of SOCS3 [72]. It has been shown that viruses with partial deletions in NS1 are attenuated, do not cause disease, and produce a protective immune response in mice [174,175]. Furthermore, influenza virus vaccines attenuated through deletion of NS1 have been shown to be immunogenic in pigs [176,177], horses [178], birds [179,180], macaques [181] and in humans (18–50 years) [154]. Aged mice are considered a relevant model to study vaccine strategies for the aged since they respond similarly immunologically [182]. Pica et al. [145] showed that vaccination of aged BalB/c mice with live attenuated influenza virus expressing the first 126 amino acids of NS1 provides protection from a single dose administered intranasally against challenge with 100 LD_50_ of wt influenza virus strain PR8. Trials have been performed in elderly human subjects where the NS1 open reading frame has been fully deleted by reverse genetics [154]. Despite the replication-deficient phenotype of the ∆NS1-H1N1, the vaccine induced local and systemic antibodies in a dose-dependant manner. The study demonstrated that the influenza strain lacking NS1 is a safe and well tolerated vaccine in humans.

### Anti-Tumour Vaccines

During tumour development it has often been reported that diminished IFN responsiveness co-evolves as a frequent genetic defect and that tumours evade their immune control by mutations frequently affecting genes involved in JAK/STAT signalling [183,184,185,186]. Studies to investigate whether such defects in tumour cells can be exploited by infection with oncolytic viruses to kill tumours have revealed a promising therapeutic strategy.

Newcastle disease virus is an avian paramyxovirus that is inherently oncolytic and tumour selective. Its tumour selectivity is thought to be due to the defective IFN response in tumour cells [187,188,189], whereas in normal cells that have a strong IFN mediated response, replication is limited [188,190]. Newcastle disease virus expresses a factor by the *V* gene (V protein) that antagonises IFN signalling and *V* gene deficient mutants grow to high titres in many tumour cells but fail to grow in normal cells [191]. Moreover, in tumour-bearing BALB/c nude mice, a *V* gene deficient mutant virus injected into the tumour replicated and spread through the tumour and effectively cleared the tumour burden [191].

Sinbis virus causes mild symptoms in humans [192] and has been shown to have considerable potential for eradicating various types of human and mouse tumours in studies using immunocompromised mice [193,194,195]. Like Newcastle disease virus, Sinbis virus is sensitive to IFN and cells defective in IFN-β production or IFN signalling are highly susceptible [196,197,198]. Hep3B cells have a low susceptibility to Sinbis virus infection, however, where STAT1 is knocked-down infection is significantly increased [199]. Furthermore, BALB/c mice bearing subcutaneous tumours in which IFNAR1 was knocked-down, showed that Sinbis virus infection significantly retarded tumour growth [199]. In addition, it was demonstrated that Sinbis virus infection also induces bystander anti-tumour immune responses. The depletion of CD8+ T cells significantly impaired the anti-tumour effect of Sinbis virus.

## 9. Safety Issues in the Use of Live Attenuated Virus Vaccines

Safety issues have recently come to light in the use of live attenuated viral vaccines where individuals have deficiencies in genes that encode factors involved in JAK/STAT signalling. A child with fatal encephalitis following inoculation with live attenuated measles, mumps and rubella (MMR) vaccine was shown to have a homozygous mutation in the IFN-α/β receptor (IFNAR2) that rendered cells from this individual unresponsive to type 1 IFN [200]. Surprisingly, the child had previously shown no evidence of heightened susceptibility to respiratory viral pathogens. In another instance, two siblings developed unusually severe viral illness. The proband developed disseminated vaccine strain measles following routine immunisation, whereas an infant brother died from an unknown viral infection [201]. The probands fibroblasts were abnormally permissive for viral replication, showed defective IFN-α/β signalling and an absence of STAT2. Sequencing subsequently revealed a mutation in the *STAT2* gene. As above for the child with a mutation in IFNAR2, individuals in the family with STAT2 deficiency remained generally healthy with no obvious defects in adaptive immunity. Findings of this nature have suggested that the systemic route of vaccine administration evades tissue-specific innate mechanisms that might otherwise complement the lack of IFN-α/β such as mucosal IFN-γ [200,202].

Attenuation of live virus by deletion of IFN antagonist genes has raised safety issues in animal studies. Cytomegalovirus encodes the protein pM27 that selectively antagonises STAT2. pM27 exploits *DNA-damage DNA-binding protein* (DDB)I-dependent ubiquitin-ligase complexes to catalyse ubiquitin conjugation of STAT2 for degradation [203]. Infection studies in SCID mice have shown that despite the considerable attenuation of murine cytomegalovirus by the deletion of *M27,* deaths were recorded at 26 days post-infection for the wt virus and 42 days post-infection for the *M27* deletion mutant virus [204].

Reducing the risk of live attenuated viruses by a combination of attenuation strategies could achieve safer vaccines and reduce the risk of revertants. Codon-pair deoptimisation (CPD) is a highly effective approach to attenuate viruses [205]. This involves recoding a viral genome by rearranging existing synonymous codons to create a sub-optimal arrangement of pairs of codons while preserving the wt virus amino acid sequence. This approach has produced striking attenuation of the PR8 influenza strain in mice without compromising protective immunity [206,207]. In addition codon-pair deoptimisation has been used to attenuate respiratory syncytial virus and an added safety feature was that the codon-pair deoptimisation strains displayed temperature sensitivity [208]. Cold adapted temperature sensitive strains of influenza virus for vaccination were first developed in the 1960s [209]. These days, the location of the temperature constraint mutations for many RNA viruses have now been mapped including respiratory syncytial virus, influenza viruses A and B, the flaviviruses dengue virus, West Nile virus and Langat virus [210]. With influenza virus it is now possible to engineer previously defined mutations into emerging pandemic strains [211].

## 10. Conclusions

In the last 20 years, there has been much discovered as to how viruses modulate IFN signalling and in many cases specific viral proteins involved have been identified. Almost all viruses have evolved strategies to combat the effects of type 1 and type 3 IFNs by antagonising the IFN signalling pathway. IFN is one of the most potent innate responses to prevent viral replication during the early stages of infection and it is clear that some of the most potent determinants of virulence are those viral encoded factors that antagonise the JAK/STAT signalling pathway. This knowledge is being exploited towards producing new generation highly attenuated recombinant vaccines that afford protective immunity. The genetic manipulations of several viruses have shown that targeting elements that block IFN signalling has major effects on viral virulence and studies on measles virus have also shown that it is possible to produce a highly attenuated virus that elicits a potent protective immune response. In the past, the means to attenuate viruses has relied on a hit-or-miss approach using conventional methods such as cell culture passaging. Nowadays, there is the potential to attenuate RNA viruses, in particular, by specific gene mutation of virulence determinants, codon-pair deoptimisation and the introduction of temperature sensitive mutations using reverse genetics. The challenge going forward for engineering live attenuated vaccines will be to reduce virulence to a level considered safe whilst retaining sufficient immunogenicity to raise a long-lasting protective immune response. With modern genetic engineering tools, highly efficacious, low cost and safe vaccines against viruses of significant global health importance may be on the horizon.
